# Flying From Hospital to Hospital: A Case of Caffeine Intoxication

**DOI:** 10.7759/cureus.8779

**Published:** 2020-06-23

**Authors:** Maria Vilela, Renato Nogueira, Joana Cunha, Ana Lima Silva, Augusto Duarte

**Affiliations:** 1 Internal Medicine, Centro Hospitalar do Médio Ave, Vila Nova de Famalicão, PRT

**Keywords:** self medication, syncopes, diuretic

## Abstract

A 54-year-old woman with a history of excess weight and active smoking presented to the emergency department (ED) due to syncope after a long flight. She reported a similar episode in the previous month, which had also occurred after a long air voyage. She presented with hypotension, dehydration, and hyperlactacidemia. The clinical team ruled out acute coronary syndrome, pulmonary embolism, and cerebrovascular accident. After clinician insistence, she reported having ingested anhydrous caffeine, an over-the-counter diuretic usually used by individuals seeking to lose weight, and a diagnosis of caffeine intoxication was made. The over-the-counter sale of this apparently innocuous substances is a rising phenomenon, and physicians should be aware of the signs of its ingestion.

## Introduction

Caffeine is used throughout the world as a social beverage and for its effects such as memory and concentration-boosting. Caffeine products are generally considered to be safe. They affect the cardiovascular and central nervous systems with positive ionotropic and chronotropic effects, as well as motor stimulatory and soothing effects. Recently, however, the use of caffeine as an over-the-counter supplement to help maximize weight loss is growing. As a result, cases involving abuse of and dependence on caffeine-containing products have become more frequent. Although uncommon, intentional or unintentional caffeine intoxication is increasing and puts people at risk of premature death [1-4].

## Case presentation

This clinical report describes the case of a 54-year-old woman living independently between the United States (US) and Portugal. She was admitted to the emergency department (ED) after being found lying prostrate and in vomit. She was overweight and an active smoker and denied having allergies or using any medications. She had amnesia with regards to the circumstances surrounding the event, but 10 minutes before being found, she had called her boyfriend to inform him that she did not feel well. She denied any change in eating habits, alcohol or tea consumption, or use of illicit substances. Hours earlier, she had taken a flight from the US to Portugal. On admission to the ED, she was alert, oriented, and cooperative; however, she was pale, dehydrated, hypotensive (blood pressure: 97/56 mmHg), and slightly tachycardic (heart rate: 104 beats/minute). Her speech was slurred, but she had no other focal neurological deficits at presentation.

The patient reported a previous similar episode, accompanied by nausea and loss of strength, occurring after a flight from Portugal to the US. She recalled feeling lethargic when she had tried to stand up quickly, before falling to the ground and vomiting. She could not remember being unconscious but said she had felt slowness of thought. Studies performed at her initial ED visit had included normal blood chemistry (BC), negative urine screening for drugs, electrocardiogram (ECG), and CT of the brain. Her lactate blood level had been 2 mmol/L. The patient had been discharged after a few hours of clinical observation. As her initial ED visit had been outside of Portugal, we did not have access to the full BC results, ECG, and CT images.

**Figure 1:**
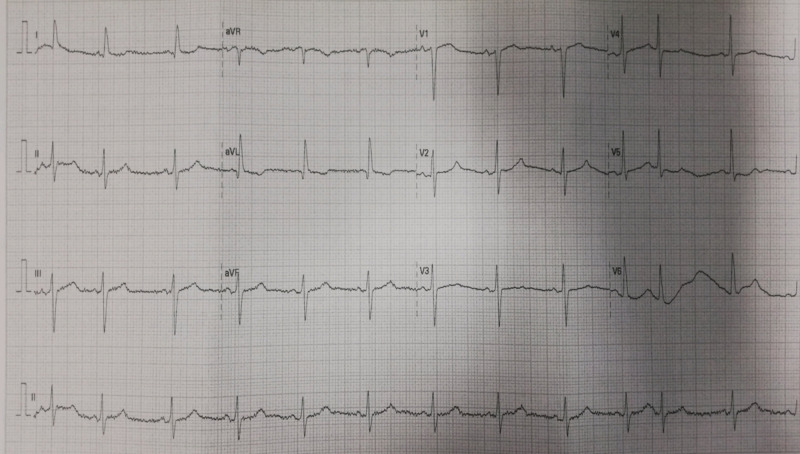
Electrocardiogram of the patient The patient presented sinus rhythm, and a heart rate of approximately 75 beats per minute, with no acute alterations

**Figure 2:**
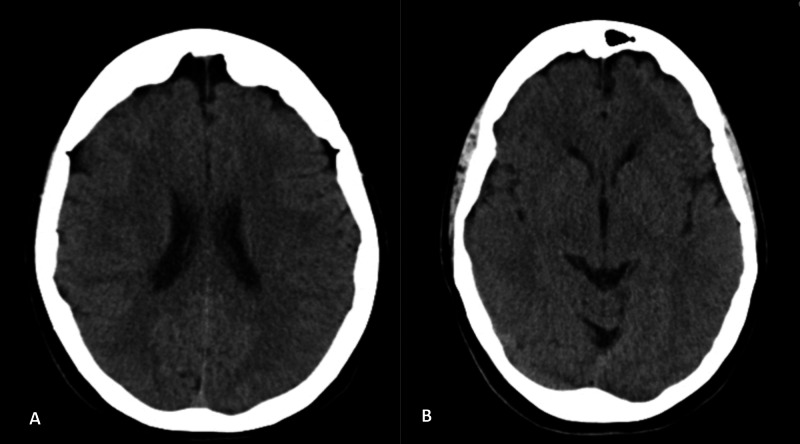
Brain CT of the patient Panel A and panel B show an expected brain CT for the age and gender of the patient, without any clinically relevant alteration CT: computed tomography

**Figure 3:**
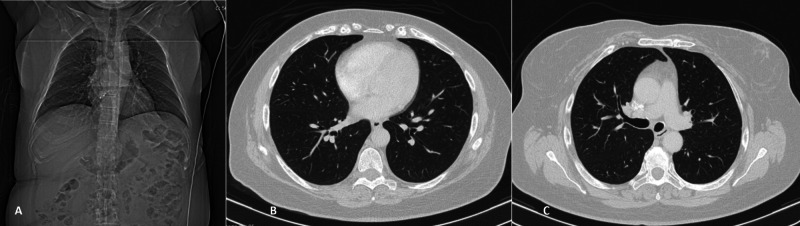
Chest angiotomography of the patient Panels A, B, and C show no thrombus in the main vessels or ischemic alterations in either lung

Upon admission to our ED, she was found to have a lactate blood level of 3.1 mmol/L, with no remarkable changes in her BC (Table 1), ECG (Figure 1), or brain CT (Figure 2). Pulmonary embolism was excluded by CT angiography (Figure 3). She was kept under observation with hemodynamic and electrocardiographic monitoring, and she remained stable throughout that period.

**Table 1:** Laboratory workup results

Analyte	Result
Hemoglobin	13.9 g/dl
Hematocrit	40.70%
Mean corpuscular volume	82.90 fL
Platelets	343 x 10^3^/µL
Leucocytes	10.96 x10^3^/µL
Neutrophils	61.50%
Lymphocytes	29.10%
Monocytes	8.20%
Eosinophils	0.20%
Basophils	0.20%
N-terminal pro-B-type natriuretic peptide	46 pg/ml
Troponin I	0.03 ng/ml
Myoglobin	20 ng/ml
Creatinine	0.69 mg/dl
Urea	28 mg/dl
Lactate dehydrogenase	162 UI/L
Alkaline phosphatase	80 U/L
Gamma-glutamyl transferase	21 U/L
Glutamic oxalacetic transaminase	27 UI/L
Glutamic pyruvic transaminase	21UI/L
Sodium	139 mEq/L
Potassium	3.5 mEq/L
C-reactive protein	0.22 mg/dl
International normalized ratio (INR)	0.96
pH	7.41
Partial pressure of oxygen (PaO2)	82 mmHg
Partial pressure of carbon dioxide (PaCO2)	39 mmHg
Bicarbonate (HCO3-)	23.9 mEq/L
Lactates	3.0
Glucose	103 mg/dl
Oxygen saturation (O2 Sat)	96%
Urine screening: amphetamines, methamphetamines, barbiturates, benzodiazepines, cocaine, methadone, opioids, cannabinoids, tricyclic antidepressants	Negative

A thorough medical history was obtained, and the patient reported taking one tablet of anhydrous caffeine (an over-the-counter diuretic sold in the US) prior to the flights mentioned above, in an attempt to prevent swelling in her lower extremities. After careful consideration, the patient was diagnosed with iatrogenic hypotension due to the ingestion of anhydrous caffeine [3,4]. The patient was discharged after a 24-hour observation period in the ED, and a medical consultation was scheduled. She was encouraged to avoid consumption of anhydrous caffeine and maintain a daily fluid intake of 1.5-2 liters.

## Discussion

Along with thorough history-taking, detection of an elevated blood lactate level is one of the few markers that can indicate caffeine intoxication [5]. Caffeine blood levels can also indicate the diagnosis; however, not all laboratories are equipped to measure this, and levels described as nontoxic in the medical literature may still cause severe health problems. There are very few reports describing the side effects of anhydrous caffeine; meanwhile, increased consumption has been reported among individuals who wish to lose weight. Although caffeine is commonly consumed worldwide, caffeine intoxication can be dangerous and, in some cases, fatal [1,6,7].

Although deaths due to caffeine are uncommon, overdoses can cause profound toxicity, resulting in tachycardia, arrhythmia, convulsions, vomiting, and coma, with some patients needing admission to the intensive care unit [1,4,6]. Death usually only occurs after large quantities of caffeine have been consumed. Nonetheless, certain individuals are at higher risk, including children, women of reproductive age, and patients with liver or cardiac disease, as they might not be able to process methylxanthines (i.e., a caffeine metabolite) fully and may die as a result of caffeine intake, even at levels well below those ordinarily considered toxic [1,2]. Caffeine intake may also be inconspicuous. Some teas or herbal remedies may contain high amounts of caffeine but do not fully disclose these concentrations. Herbal remedies, teas, and kitchen food items could represent a problem if ingested in large amounts or in concentrated fashion (such as caffeine tablets). The wide variety of symptoms presented, ranging from mild tachycardia to convulsions or even coma, can also be misleading. The clinical cases reported in medical literature focus on the more severe cases, and the real incidence of caffeine intoxication may be greatly underestimated.

## Conclusions

In the absence of strict regulations, the consumption of caffeinated substances could cause a high number of ED admissions. The unrestricted sale of seemingly innocuous substances is a phenomenon on the rise, and all clinicians should be alert to this problem. Clinicians need to consider these new drugs in their diagnoses and readily investigate and treat possible intoxications. The most important tool clinicians have at their disposal is a thorough anamnesis, and this case provides a paramount example of clinicians utilizing that tool to outstanding effect.
